# Implementation of constrained swept synthetic aperture using a mechanical fixture

**DOI:** 10.3390/app13084797

**Published:** 2023-04-11

**Authors:** Nick Bottenus

**Affiliations:** 1 Department of Mechanical Engineering, University of Colorado Boulder, Boulder, CO 80516, USA

**Keywords:** Beamforming, Synthetic aperture, Image quality, Speckle tracking, Displacement estimation

## Abstract

Resolution and target detectability in ultrasound imaging are directly tied to the size of the imaging array. This is especially important for imaging at depth, such as in the detection and diagnosis of hepatocellular carcinoma and other lesions in the liver. Swept synthetic aperture (SSA) imaging has shown promise for building large effective apertures from small physical arrays using motion, but has required bulky fixtures and external motion tracking for precise positioning. In this study we present an approach that constrains the transducer motion with a simple linear sliding fixture and estimates motion from the ultrasound data itself using either speckle tracking or channel correlation. We demonstrate in simulation and phantom experiments the ability of both techniques to accurately estimate lateral transducer motion and form SSA images with improved resolution and target detectability. We observed errors under 83 *μ*m across a 50 mm sweep in simulation and found improvements of up to 61% in resolution and up to 33% in lesion detectability experimentally even imaging through ex vivo tissue layers. This approach will increase the accessibility of SSA imaging and allow us to test its use in clinical settings.

## 1. Introduction

Ultrasound imaging is widely used for both screening and diagnosis in soft tissues due to advantages in cost, safety, and portability over competing imaging technologies [1-3] especially with the advent of portable devices [4]. Image contrast is based on differences in the acoustic impedance of tissues, providing good visualization of cysts, tumors, vessels, and similar structures. For example, ultrasound has long been the primary screening tool for hepatocellular carcinoma (HCC). However, target detectability in images is based on the combination of contrast and resolution [5] – the latter a particular weakness of ultrasound imaging. In HCC screening a recent meta-analysis found only 45% of early-stage HCCs were detected by ultrasound alone [6], bringing into question its utility without added blood tests or other imaging modalities.

While axial resolution is related to the length of the transmitted imaging pulse (often approximately a wavelength, *λ*), lateral resolution is based on the wavelength and the f-number, *F* = *z/D* for depth *z* and imaging aperture size *D*. This leads to anisotropic resolution that varies drastically through depth. In low-contrast lesions a loss of resolution blurs lesion boundaries and fills off-axis information from surrounding tissues into the hypoechoic spaces. In abdominal or obstetric imaging where imaging depths can exceed 10–20 cm, especially in obese patients, lateral resolution can be worsened to several millimeters. This makes characterization of very small suspected HCC lesions (< 1 cm) impossible until further growth has occurred [7], lessening the advantages of early detection. Current LI-RADS protocols suggest the alternatives of contrast-enhanced ultrasound or CT/MRI for diagnosis and staging of HCC [8,9], with standard B-mode imaging unable to provide clear tumor size measurement.

While the imaging frequency (and wavelength) of ultrasound is limited by the required penetration, with higher attenuation at higher frequencies, the aperture size is fundamentally only limited by the available acoustic window. A standard curvilinear imaging array used in abdominal applications has a total aperture size around 5–6 cm, although these elements point radially outward to increase field of view and result in an even smaller coherent active aperture size. We have recently demonstrated the value of increasing aperture size to improve lateral resolution and detectability at depth in simulation [10], *ex vivo* experiment [11], and *in vivo* imaging[12] even in the presence of image-degrading clutter effects. Multi-transducer experiments have demonstrated improvements in resolution that scale with the effective aperture extent [13-15] and provided enhanced visualization of *in vivo* liver and kidney structures.

Despite the promise of large aperture imaging strategies, there are practical barriers to the implementation of such systems. The cost, weight, and electronics complexity of arrays scales with the number of array elements required, especially when considering 2-D array systems [16]. Point of care systems are unlikely to implement large apertures as they are designed to be low cost and highly portable, often imposing data bandwidth constraints on the system as well. We have previously introduced a reduced complexity method for large aperture imaging termed “swept synthetic aperture” (SSA) imaging [12,17] that may be more widely applicable, using motion of the ultrasound array to form a larger effective coherent array than the physical footprint of the device. This is similar to a monostatic imaging approach [18] with the advantages of improved signal-to-noise ratio and signal redundancy provided by an imaging array. Motion has been extensively used in ultrasound to form larger fields of view [19] and 3-D images [20], both using external tracking systems [21,22] and image-based motion estimation [23,24] approaches, but these methods simply stitch together B-mode images rather than coherently combining data for higher resolution.

The SSA approach requires precise array position and orientation knowledge during the sweep to perform synthetic aperture beamforming of the data from each array position to a common imaging field of view. It is known that synthetic aperture imaging is especially sensitive to errors in the axial direction, requiring sub-wavelength precision [25]. SSA imaging similarly requires sub-wavelength jitter error (random position-to-position variation), but is much less sensitive to calibration errors (a fixed error in position/orientation) [12]. Initial SSA investigations found varying impacts of translation and rotation errors in different directions, with approximately equal impacts of translation in the axial and lateral dimensions (an achieved reconstruction precision of 0.1 mm given jitter error around *λ*/5 or calibration error around 2*λ* for both dimensions). Previous approaches to SSA imaging have used either a robotic positioning system or a mechanical fixture with external position measurement to achieve this required precision[12,17]. These approaches also required precise spatial calibration to relate the position measurement to the actual position and orientation of the array. These requirements significantly limit the translation of the approach to clinical settings.

In this work we present an alternative SSA approach that makes use of the ultrasound array signals themselves to estimate transducer motion with sufficient precision for synthetic aperture beamforming. To achieve this we constrain the motion to one degree of freedom using a simple external fixture with no measurement capabilities to simplify the estimation problem. We characterize two methods of estimation – lateral speckle tracking and channel signal correlation – for use in this application. Preliminary phantom results were presented in a conference proceedings [26] and are expanded here in addition to simulation study.

## 2. Materials and Methods

### 2.1. Pulse sequencing

Proper pulse sequencing is essential for successful SSA imaging using motion tracking. The motion tracking methods below assume there is only slight decorrelation between successive data sets representing a small motion of the array. For a focused transmission to a point in space, a translating array produces decorrelation relative to its length [27]. Increasing decorrelation is associated with increasing variance of displacement estimates [28]. To maximize the correlation, unsteered (0 degree) plane waves were transmitted and echo data were collected on all array elements. This plane wave can be viewed as a relatively constant transmit field, with only small differences near the edges of the array extent.

However, the plane wave transmission is a poor choice for SSA as the field of view only overlaps over a small range of aperture positions[26]. The transmit aperture remains constant (unfocused) and the receive aperture can only effectively double in length due to the finite width of the plane wave. Instead we use a diverging wave to produce a broad transmit field that shifts with position of the array. This transmit signal creates rapidly decorrelating echoes with translation, a poor choice for displacement estimation but ideal for maximizing the extent of both the effective transmit and receive apertures over a sweep. The effective aperture length reached is calculated as the average of the swept transmit and receive apertures.

**Figure 1.**
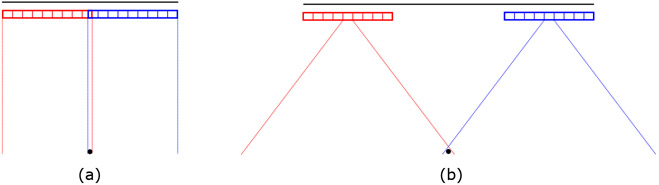
Illustration of transmit geometries used for correlation-based SSA imaging showing two array positions imaging a target (black circle). Black lines above the array show the synthetic receive array length. (a) Plane wave transmission produces highly correlated echoes for motion tracking but limits spatial overlap and does not produce an improved synthetic transmit aperture. (b) Diverging wave transmission produces long effective transmit and receive arrays for high resolution due to large spatial overlap at depth but has low echo correlation for motion tracking.

**Figure 2.**
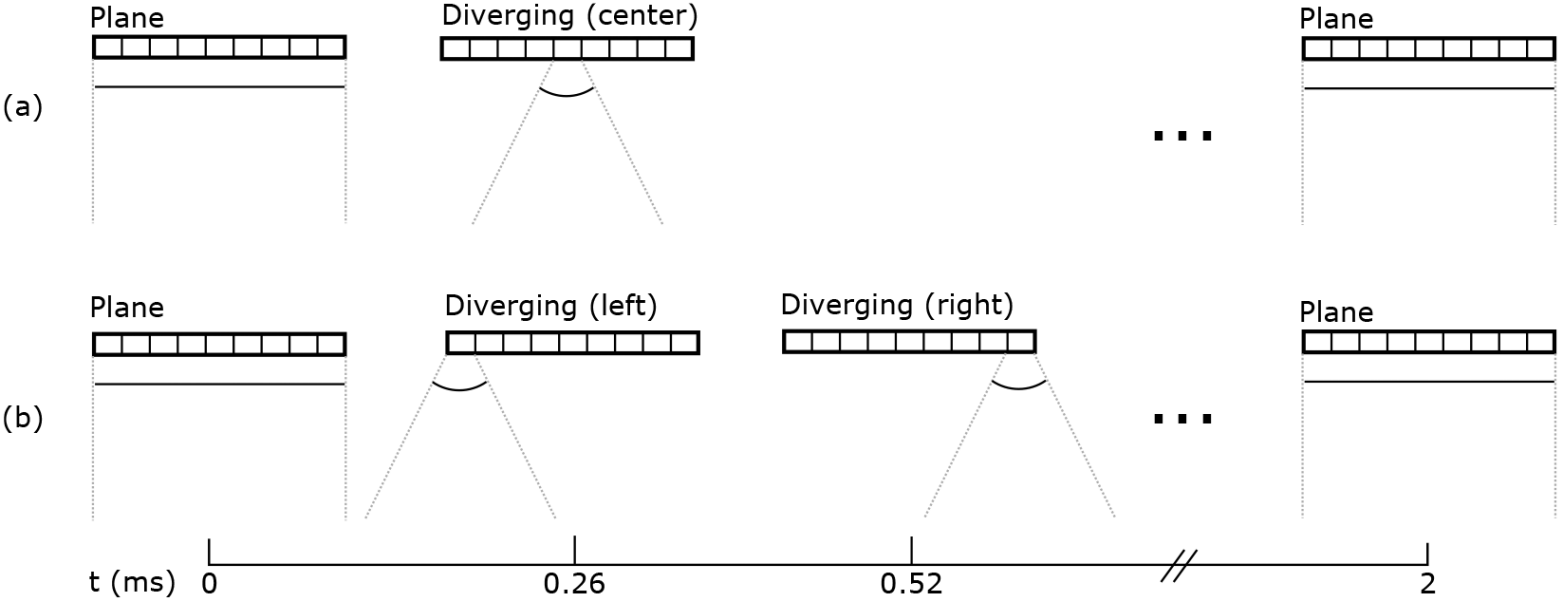
Sample pulse sequences for correlation-based swept synthetic aperture imaging using 500 Hz frame rate and 260 *μ*s between pulses to allow propagation and data acquisition. The ellipses represent a gap between transmissions. (a) Alternating plane and diverging waves, with plane waves to provide highly correlated data and diverging waves to provide a wide acceptance angle for coherent combination. (b) Diverging waves transmitted from each edge instead of the center to maximize the effective transmit aperture extent.

These transmit geometries and their trade-offs are illustrated over a sweep of the transducer in Fig. 1. Assuming a constant transducer motion that covers 10 cm in 1 second, a pulse repetition frequency of even 1 kHz would provide spatial sampling of 0.1 mm which is oversampled compared to past work [12]. We expect that even if the maximum velocity is increased due to acceleration required at the beginning and end of the sweep the displacement will remain small, on the order of the element pitch. These transmissions can therefore be interleaved into a single sequence to provide highly correlated data for displacement estimation and decorrelated data for synthetic aperture imaging, as shown in Fig. 2. The simplest sequence would have alternating plane waves and diverging waves, repeating at the chosen repetition frequency. An improved sequence would use two diverging waves, one from either end of the array, with the plane wave. This extends the effective transmit aperture for the SSA image by an entire transducer length (half in each direction at the ends of the sweep). This diagram shows the timing used in this paper with 260 *μ*s between pulses within the sequence and an overall frame rate of 500 Hz. While in these diagrams the diverging wave is shown produced by a single element, it is also possible to use subapertures to create diverging virtual sources [29] with improved signal-to-noise ratio (SNR).

### 2.2. Simulation

We used Field II[30,31] to simulate transducer motion for mixed target phantoms to study the optimization of estimation parameters. We simulated the P4-2v phased array transducer with center frequency 3 MHz, 0.3 mm element pitch, 64 elements, and 80% bandwidth. Simulations were performed at 120 MHz sampling rate and channel data were stored at 20 MHz output sampling frequency. We simulated 120 mm (axial) x 200 mm (lateral) x 2.5 mm (elevation) speckle-generating material at 15 scatterers per resolution cell at the expected SSA resolution (approximately equivalent to that of a 7 cm array), with a 4-mm radius anechoic lesion at 6 cm depth and point target at 10 cm depth. We repeated simulations for 10 different realizations of randomly positioned and weighted scatterers. We performed four sets of simulations:

1. Reference image – Multistatic acquisition (each transmit/receive element pair) from a single array position generates a synthetically focused baseline image without the SSA technique.
2. Subsample estimation sweep – Simulations of plane wave transmission and single-element diverging transmission (from the center of the array) were produced from fine displacements up to a single element pitch in 0.03 mm steps.
3. Large displacement estimation sweep – Simulations of plane wave transmission and single-element diverging transmission were produced from larger displacements as would be found in a 5 cm SSA sweep in steps of 0.1 mm.
4. Equivalent image – Full synthetic aperture acquisition from a fully populated 7 cm long array produces a similar image to the 5 cm SSA approach when the active transmit aperture is limited to 5 cm.

### 2.3. Phantom acquisition

#### 2.3.1 Translation stage

Constrained SSA was achieved with a known ground truth motion profile using a Newport UTM100 linear translation stage (Newport, Irvine, CA). The P4-2v transducer was attached to the stage using a custom molded holder such that the array direction was aligned with the direction of motion. The stage was programmed over a serial communication interface and motion was synchronized with the imaging pulse sequence described below.

#### 2.3.2. Slide device

**Figure 3.**
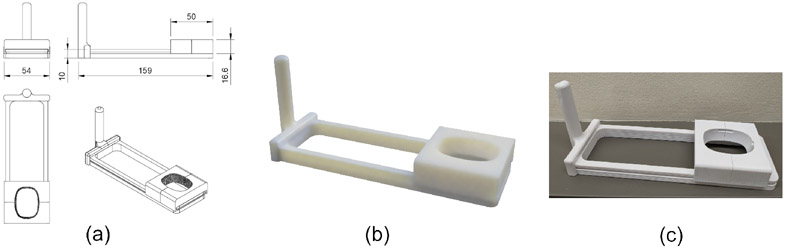
Mechanical slide fixture allowing 1-D movement along the array dimension. (a) Dimensioned diagram (units mm), (b) Rendering, (c) 3-D printed device.

Constrained SSA was achieved without robotic control or external tracking using a 3-D printed slide fixture designed for the P4-2v ultrasound transducer shown in Fig. 3. A track was designed allowing up to 10 cm motion (center to center) of the array along a linear path. A sliding block was designed to ride in a cutout track, fitting tightly to prevent tilting or twisting of the array but loose enough to slide freely when moved by hand. A 3-D scan of the P4-2v transducer was used to create a hole the appropriate size and shape in the sliding piece, enlarged to allow for the dimension tolerance of the printed PLA filament. The sliding piece was divided into two pieces to allow for easy insertion and removal of the transducer. The sweep is oriented along the array elements (i.e. within the imaging plane). A handle was added to assist in holding the device still during the transducer sweep but is removable. The pieces were printed using a standard Lulzbot Taz 5 desktop 3D printer (Aleph Objects, Loveland, CO).

#### 2.3.3. Data acquisition

Data were acquired using the Verasonics Vantage 256 research ultrasound scanner (Verasonics Inc, Redmond, WA) and P4-2v phased array transducer. This is a small phased array transducer with 0.3 mm element pitch and 64 elements. Three studies were conducted:

1. The imaging target was the ATS 549 tissue mimicking phantom (CIRS, Norfolk, VA) and the transducer was attached to the translation stage as described above. The transducer was swept over 5 cm in 0.033 mm steps, alternating between plane wave transmission and left/right diverging transmissions (virtual source, 11 elements with f-number −0.75) in that order to mimic a continuous sweep with spacing of 0.1 mm between repetitions.
2. The transducer was used with the linear slide device on the ATS 549 phantom. The phantom was positioned such that point targets were located below lesion targets and both were visible in the image. The transducer was coupled with gel and the device was held steady on the phantom surface by hand. The transducer was swept along the track covering approximately 5 cm in 1 second, although temporal dynamics and extent varied from sweep to sweep. During the sweep transmissions were alternated as described in Fig. 2(b) at an overall pulse repetition frequency of 500 Hz.
3. The manual sweep was repeated with an *ex vivo* tissue sample between the transducer and an ATS 539 phantom. The phantom was positioned so that lesion targets were located below point targets and both were visible in the image. Three different store-bought meats were used to study whether the intervening layer affected motion tracking. Pork chop (12 mm thick), beef loin (10 mm thick) and chicken breast (9-19 mm thick) were used as varying models. Samples were prepared to be wider than the slide device so it rested entirely on top of the sample. The degassed samples were placed on top of plastic wrap and all layers were coupled with gel. The same sweep sequence was used as above.

In each case channel radio frequency (RF) data were stored for offline processing. For each, a reference image (i.e. no sweep) was also acquired using steered plane waves from −45 to 45 degrees.

### 2.4. Motion estimation methods

**Figure 4.**
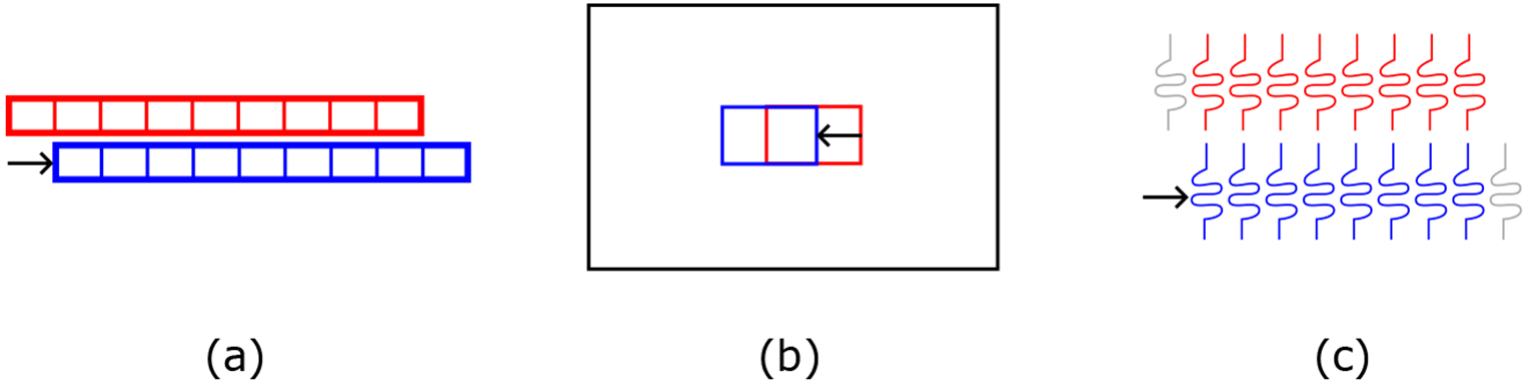
(a) Assuming low decorrelation over small displacements, motion of the array (here by one element pitch) can be estimated using a cross-correlation search between plane wave transmissions. (b) Speckle tracking uses a 1-D search of an image kernel between a reference and target image over a field of view relative to each array position. (c) Channel correlation compares shifted pairs of raw channel signals with varying offsets, reducing the active subaperture with increasing shifts.

Because transducer motion is constrained to occur within the imaging plane it should be visible in the imaging data as a contrary motion of the imaging targets. Although ultrasound is best suited to motion estimation in the beam direction[32,33], it is possible to estimate motion in the transverse (lateral) direction using speckle tracking[34] or transverse oscillation[35,36] approaches. We additionally present an alternative approach that is well suited to the constrained motion problem. These approaches are shown in Fig. 4. In both cases we use the same recorded data from plane wave transmissions to maintain an approximately constant transmit field and measure motion of the receiving array (using the diverging wave data only for image formation). While it should be possible to measure displacement from a spatially varying transmit field as well, that would result in additional decorrelation[37] and is outside the scope of this paper.

#### 2.4.1. Speckle tracking

Speckle tracking uses focused images (radio frequency or envelope detected, channel or beamsum) with cross-correlation to track motion between pairs of frames. It is assumed that the speckle patterns remain largely correlated for small displacements of the receiving array[27] and can be matched. For our lateral estimation, we performed envelope detection on the beamsum data to reduce axial variations and performed all operations on a Cartesian grid. Given a 2-D reference region (or kernel) from the first plane wave image, we used normalized cross correlation to search laterally in the second plane wave image for the best matching position as shown in Fig. 4(b). The observed motion in the image is opposite the motion of the transducer. The parameters of this estimation (kernel size and depth) are explored in the simulation section. For phantom imaging, the kernel and search region were chosen to be 10 mm x 10 mm at a depth of 20 mm (30 mm for cases with added tissue), with a 2.5 mm lateral search region in each direction.

#### 2.4.2. Channel correlation

While speckle tracking is effective, it is a tool designed for varying motion estimation throughout a field of view. We introduce a more direct estimation tool for the problem of transducer displacement. Channel correlation relies on the idea that for small motions there will be subapertures from the initial and displaced position that spatially overlap (possibly with some sub-pitch offset). The backscattered echoes observed by these subapertures should be highly correlated given a relatively constant transmit field. We can therefore search for transducer motion by correlating the recorded channel data from these subapertures without the need for focusing or image formation. We study subsets of channels as shown in Fig. 4(c), removing channels from opposing ends of the array for the initial and displaced data sets to increase the search offset. We used the same estimation depth and axial kernel size as in speckle tracking (using the raw echo signals rather than beamformed images) and searched up to offsets of 10 elements (3 mm).

#### 2.4.3. Subsample and multi-lag estimation

For both methods it is necessary to perform subsample estimation to precisely track motions between frames. For speckle tracking, the beamformed lateral pixel sampling determines the quantization of motion estimates. For channel tracking, estimates are quantized to the element pitch. While it is possible to adjust the choice of lateral pixel sampling to improve estimation accuracy, that is not possible for the array elements used in channel correlation. Instead we fit the correlation curve as a function of displacement (lag) to a model allowing us to estimate the location of the peak correlation and the peak correlation value[38]. We chose to use the iterative reconstructive interpolation method for all subsample estimation in this work, although this requires a strictly bandlimited signal and may produce errors for spatially undersampled (aliased) signals. In those cases a polynomial fit may produce a better result.

In order to reduce both noise and quantization error we implemented a multi-lag estimation scheme using displacement estimates from multiple overlapping pairs of frames[39]. We constructed a measurement matrix H where each row represents the difference in position between two frames, up to 10 frames apart, and the estimated displacement is stored in the matrix Y. The peak normalized cross-correlation coefficient was used to weight each observation with the matrix W, and the individual frame positions $\hat{X}$ were estimated using the weighted least squares solution:

(1) $$\hat{X}={\left(H^{T}WH\right)}^{-1}H^{T}WY$$

While we chose a maximum separation of 10 frames, if computation time were not an issue we could continue to extend this matrix with more distant pairs until the correlation coefficient indicated that the estimates were no longer useful.

Code for these methods is made available at https://github.com/bottenuslab/lateral_transducer_tracking (DOI 10.5281/zenodo.7682384).

### 2.5. Image reconstruction and analysis

Reference images (i.e. no sweep) were formed using the plane wave imaging data with standard plane wave focusing methods[40]. Swept synthetic aperture images were formed using standard diverging wave focusing methods with varying transmit and receive aperture positions[12] according to either the translation stage (or simulated) position or the estimated motion (from plane wave transmissions). In both cases, diverging wave data were resampled to retain frames at roughly 0.5 mm spatial intervals, reducing the computation required for image formation and avoiding artifacts due to the varying temporal profiles of the manual sweeps. It was assumed that all motion occurred along the lateral axis within the imaging plane and there was no rotation of the transducer. For the interleaved acquisition sequence the motion estimates from the plane wave frames were interpolated to provide estimates of positions for both the left and right diverging wave transmissions. The focused images from the left and right transmissions were coherently combined, and frames were weighted with a Tukey window to reduce high spatial frequency components of the synthesized transmit aperture present in the SSA image compared to a fully sampled large array[12].

Image analysis was performed using standard methods for resolution and lesion detectability measurement. The lateral point spread function was displayed using a slice through the peak of the point target, with the highest value normalized to 0 dB. Lesion detectability was quantified using the generalized contrast-to-noise ratio (gCNR):

(2) $$\text{gCNR}=1-\int_{-\infty }^{\infty }\text{min}\{f(x)g(x)\}\;dx,$$

where f and g are the normalized histograms of two image regions[41]. Histogram matching was applied to images across different conditions to visually match speckle background appearance (mean and variance) despite variation in peak value across images[42].

## 3. Results

### 3.1. Simulation

#### 3.1.1. Displacement estimation

**Figure 5.**
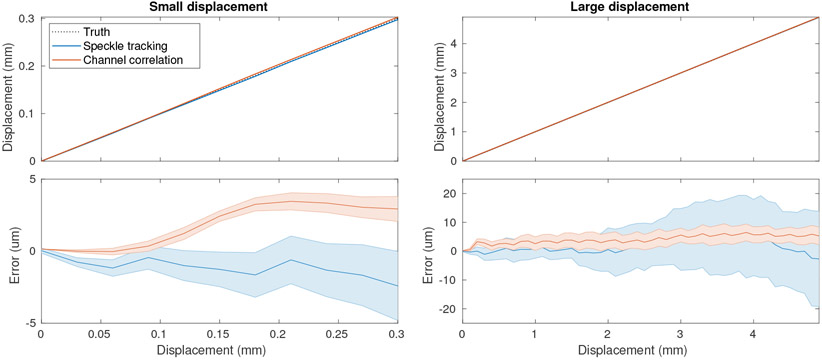
Demonstration of speckle tracking and channel correlation for displacement estimation using individual estimates on pairs of frames. Estimates performed with a 10 mm x 10 mm kernel. (left) Displacement estimates and error over a short distance, less than one element pitch. Error bars represent 10 speckle realizations. A ±2.5 mm search region was used. (right) Estimates and error over larger distances, up to 5 mm. A ±7.5 mm search region was used. Errors depend on choice of sampling, kernels, and subsample estimation method.

We first investigated lateral displacement estimation using the two proposed techniques – speckle tracking and channel correlation – in simulation. Fig. 5 explores the error in estimation faced over varying displacement distances using individual pairs of frames (i.e. no multi-lag estimation). Over short distance scales, up to the element pitch, the two methods demonstrate different behaviors. Speckle tracking (lateral pixel spacing *λ*/4 = 0.13 mm) shows an oscillating error possibly related to the acoustic resolution, pixel spacing, and subsample estimation method chosen (iterative reconstruction). We expect that this behavior could change for varying choices of these parameters. Channel correlation shows a much smoother performance because the channel spacing is much coarser (0.3 mm) and the estimate does not use focused signals. However, it should be noted that in both cases error is limited to less than 5 *μ*m, or *λ*/100.

Even over larger distances, up to 5 mm in Fig. 5, we see good performance of both methods. Channel correlation shows better standard deviation than speckle tracking over this distance, although both are small relative to the wavelength and have a mean near zero. We also see cyclic errors in the estimate here, with a period of one element pitch (0.3 mm) for channel correlation. These data suggest that we can use multi-lag estimation representing both small and large displacements to further improve our estimates, and can tolerate fairly fast transducer velocities that will lead to several millimeters of displacement between observations.

**Figure 6.**
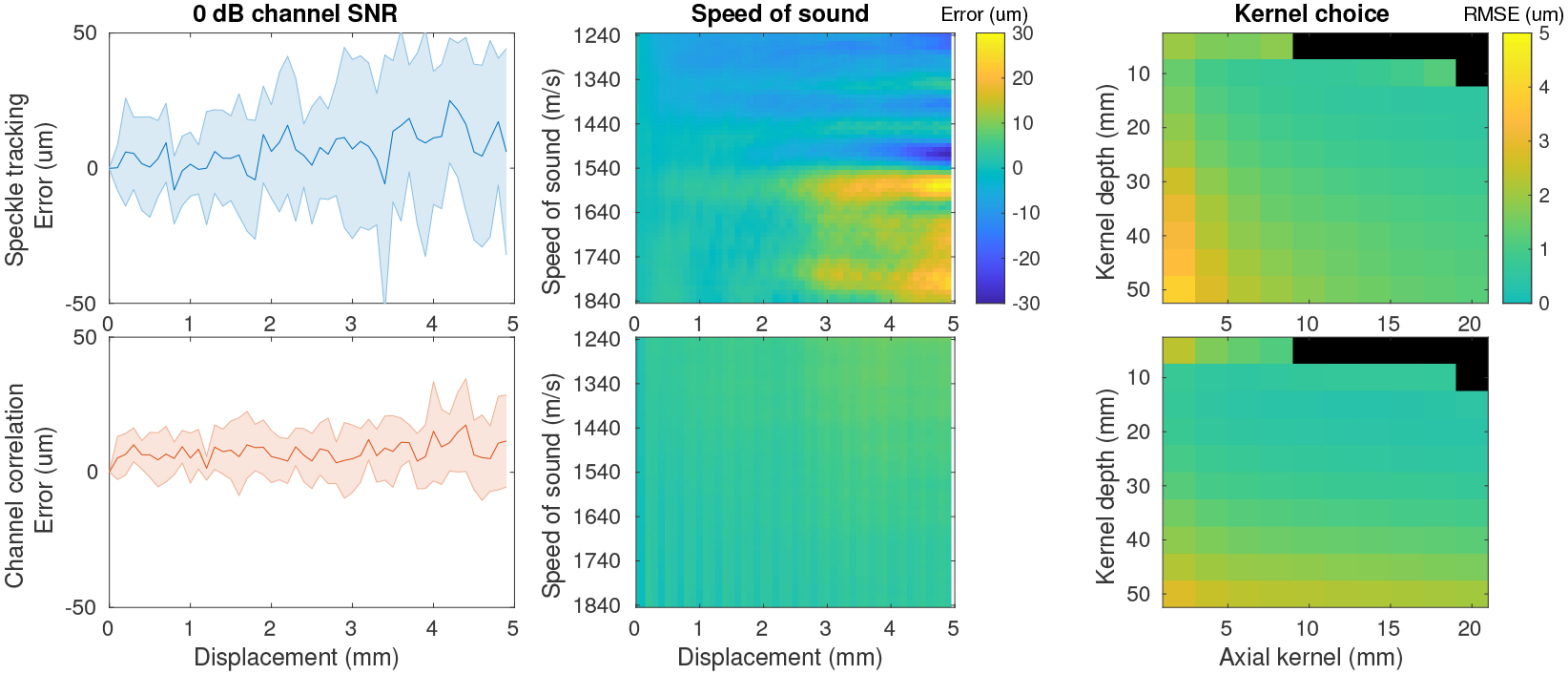
Challenges to and optimization of estimates in simulation. (left) Additive channel noise reduces accuracy compared to Fig. 5. (middle) Errors in assumed speed of sound affects speckle tracking accuracy but not channel correlation (true speed of sound 1540 m/s). (right) Speckle tracking is more sensitive than channel correlation to choice of kernel location and depth. (black indicates regions where the chosen kernel extended beyond the data/image extent.)

We sought to study challenges that these methods may face in practice such as noise and sound speed error and to determine the best estimation parameters to use, with results shown in Fig. 6. First, we explored the impact of bandlimited additive Gaussian noise on the estimation. Reducing channel SNR to 0 dB (measured at the same depth used for displacement estimation) resulted in additional variance in the estimates compared to what was seen in Fig. 5, but the estimates remain reasonably unbiased and show standard deviation less than 50*μ*m over the 5 mm distance. Second, we introduced error to the speed of sound used in estimation (including image formation for speckle tracking), varying it from 1240 m/s to 1840 m/s compared to the correct 1540 m/s. This error led to slightly varying depths of estimation and effective kernel sizes but largely had no effect on the channel correlation approach. The speckle tracking approach, due to the distortion of the images produced, showed average positive and negative estimation errors at larger distances up to approximately 30 *μ*m. This bias is likely the result of the stretched speckle pattern used in correlation. Finally, we studied the impact of kernel choice on the estimates. We varied the depth of estimation and the axial kernel extent for both methods and calculated the root mean square error of the estimates (all showed roughly linear profiles as in previous cases). We observed some increase in error at larger depths and reduction of error with a larger axial kernel. We also found greater sensitivity and larger errors for speckle tracking, but both methods had average errors under 5 *μ*m. While we observed better performance for channel correlation across these tests, it should be noted that we do not expect that the errors observed in either case are sufficient to degrade SSA imaging performance.

#### 3.1.2. Imaging results

**Figure 7.**
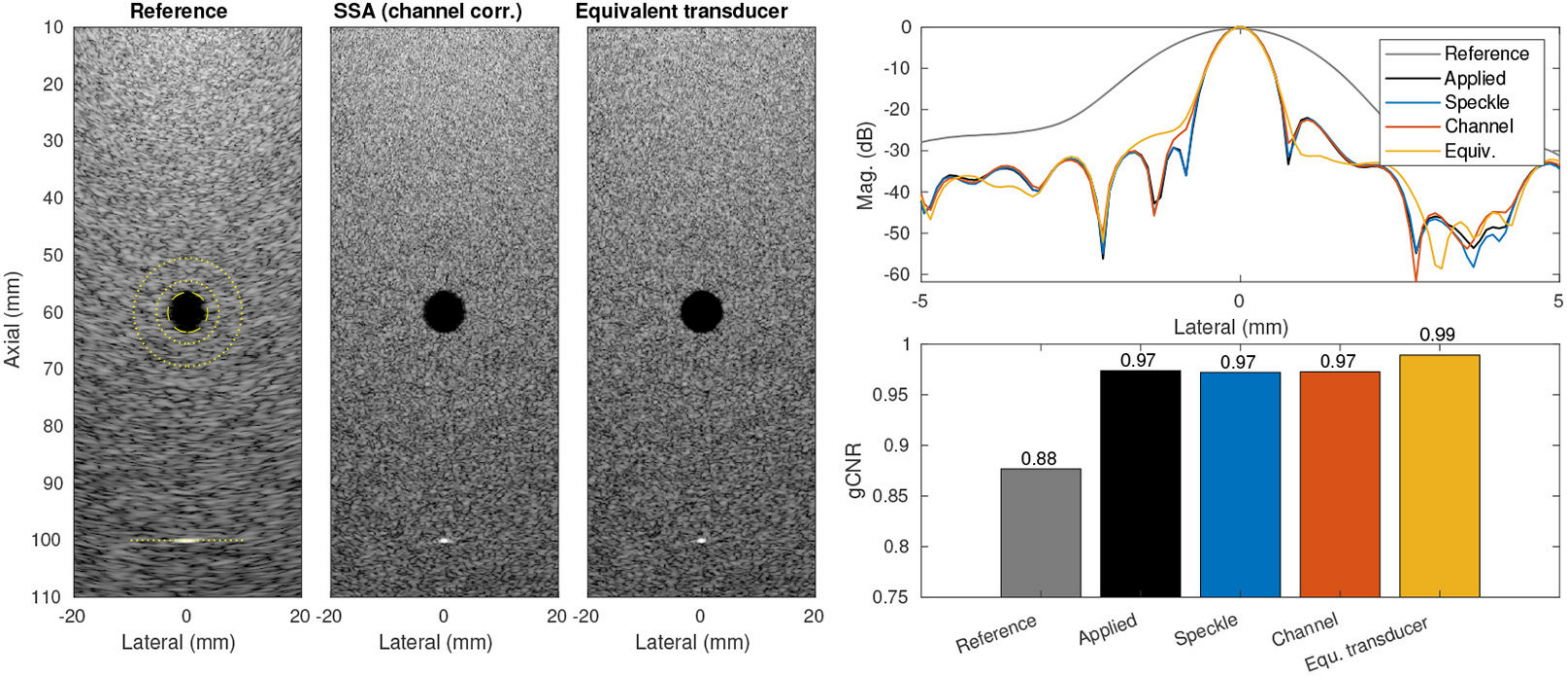
Simulation comparison of reference image, SSA images, and equivalent transducer matched to the effective length of the SSA acquisition. (left) Images for reference, channel correlation estimated SSA, and equivalent transducer, shown with 50 dB dynamic range and ROIs indicated for lesion and point. (right top) Lateral PSF for point at 10 cm depth. (right bottom) gCNR for anechoic lesion target located at 6 cm depth.

We used the estimates from both methods to produce SSA images over a simulated 50 mm transducer sweep of the 19.2 mm array. Fig. 7 shows a reference image produced by synthetic aperture imaging (coherent plane wave compounding) from a single transducer position. Full-width at half-maximum (FWHM) point target resolution at 100 mm depth was measured to be 2.20 mm. We observed very similar performance for the two estimation methods compared to image formation using the true applied motion profile, improving the FWHM to 0.70 mm in both speckle tracking and channel correlation, matching the ideal case. The maximum errors in displacement estimation for speckle tracking and channel correlation were 12 *μ*m and 83 *μ*m respectively. We also compared these results to a fully sampled large array with a 70 mm receive aperture and 50 mm transmit aperture that is roughly equivalent to the effective array formed by SSA. For that array we found a FWHM of 0.70 mm as well. We expect to observe some differences due to the spatial frequency content differences between a fully sampled array and SSA effective aperture, reflected in a trade-off between resolution and side lobe performance. We also measured gCNR for the anechoic lesion in the images and found similar improvements across the cases, with SSA improving gCNR and the estimated motion cases performing close to the ideal cases (slightly lower than with the equivalent large array).

### 3.2. Phantom

#### 3.2.1. Translation stage

**Figure 8.**
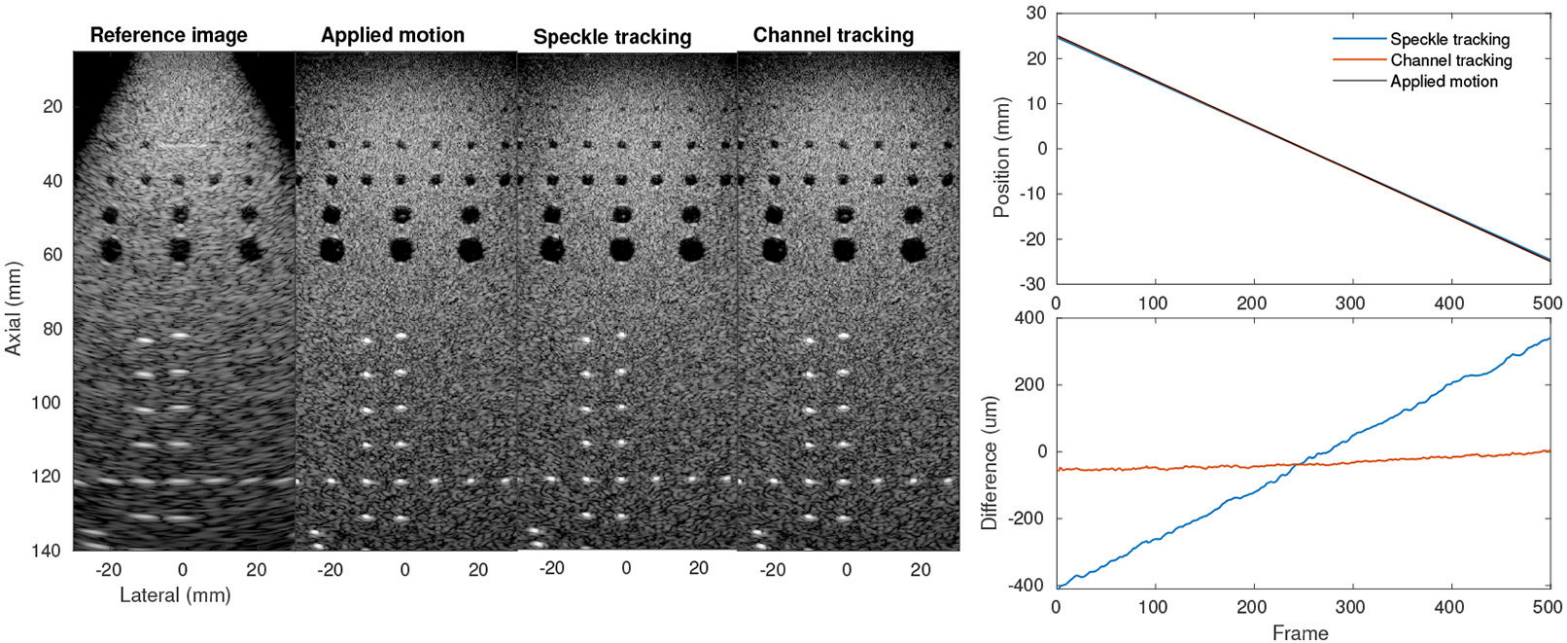
Phantom experiment with lateral motion applied by translation stage. (left) SSA images show significant resolution improvements compared to the reference image, produced by a 19.2 mm array (50 dB dynamic range). (right) Estimated motion over 50 mm translation shows strong agreement between speckle and channel tracking estimates compared to the applied motion, with error under 400 *μ*m.

We studied the application of the two estimation techniques to SSA imaging in a series of phantom experiments. Fig. 8 shows the initial experiment comparing the estimates to a known motion as applied by a translation stage. Both methods were able to accurately reproduce the applied motion profile over a span of 50 mm with maximum error for the speckle tracking method around 400 *μ*m and for the channel correlation method under 50 *μ*m. Interestingly, the estimate for channel correlation is biased, not showing the usual linear trend across the sweep. This may be due to the fixed step size creating the same slightly biased error on each step according to the subsample estimation strategy, meaning it would not be seen in freehand sweeps with varying step sizes.

**Figure 9.**
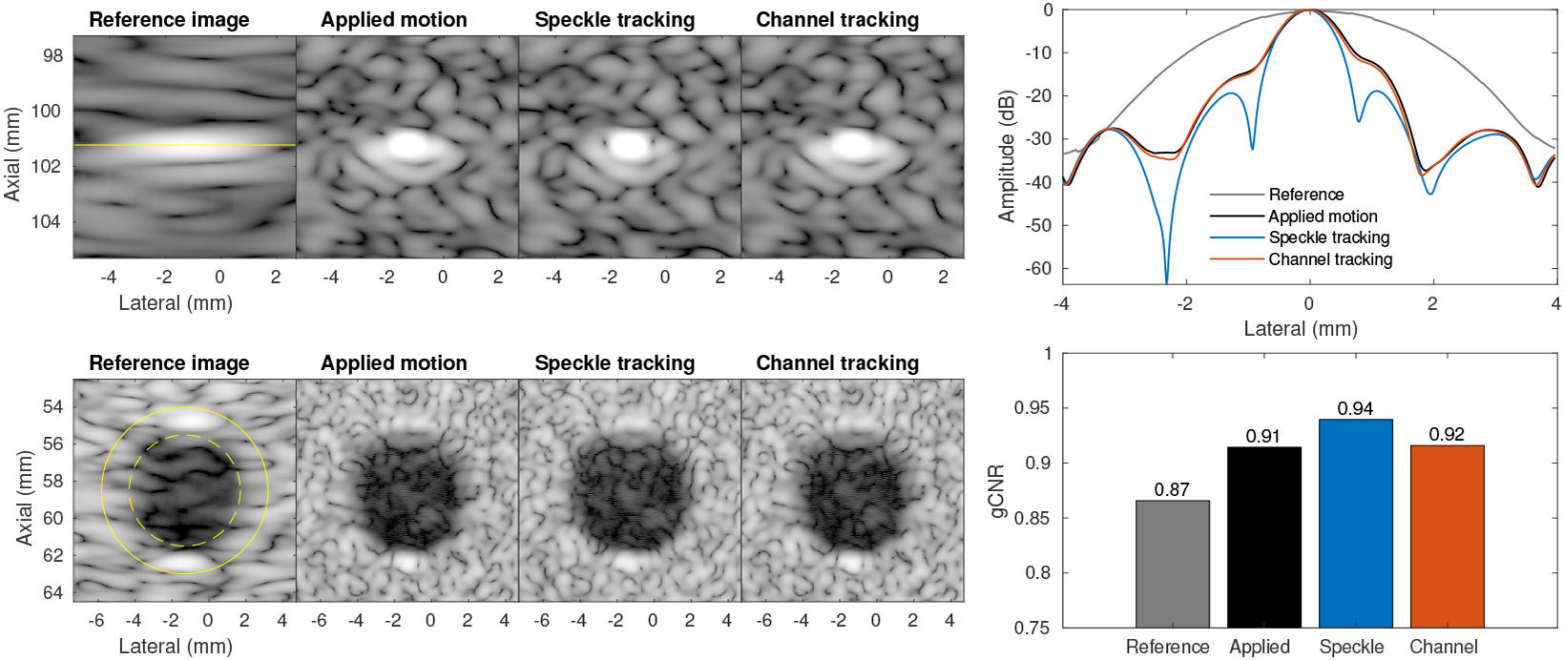
(top) Point target images corresponding to the central point target at depth 100 mm from Fig. 8. Yellow line indicates the depth for the lateral PSF. (bottom) Lesion target images corresponding to the central large lesion target at depth 60 mm from Fig. 8. Yellow circles indicate ROIs inside the dashed circle and outside the solid circle for gCNR calculation. All images show 50 dB dynamic range. Both metrics show comparable performance for the two motion tracking techniques and similar performance to the applied motion case, with slight improvements using speckle tracking.

Both strategies produced SSA images comparable to the ideal case. This is further explored in Fig. 9 by quantifying the FWHM resolution of a point target and gCNR of a lesion target. Resolution was improved from 3.22 mm in the reference case to 1.04 mm, 0.93 mm, and 1.03 mm in the applied motion, speckle tracking, and channel correlation cases respectively. It should be noted that the PSF for the speckle tracked case is noticeably improved from even the applied motion case. It is possible that the translation stage transducer mount was slightly misaligned from the direction of stage motion and that the speckle tracking technique, directly measuring target motion, compensated for this error. This seems plausible as the channel tracking result shows very small improvements over the applied motion case as well, although not as significantly as with the speckle tracking approach. Lesion detectability measured by gCNR similarly confirms these improvements, with the speckle tracked SSA case producing the clearest lesion and lesion boundary but all SSA cases improving on the reference case.

#### 3.2.2. Linear fixture

**Figure 10.**
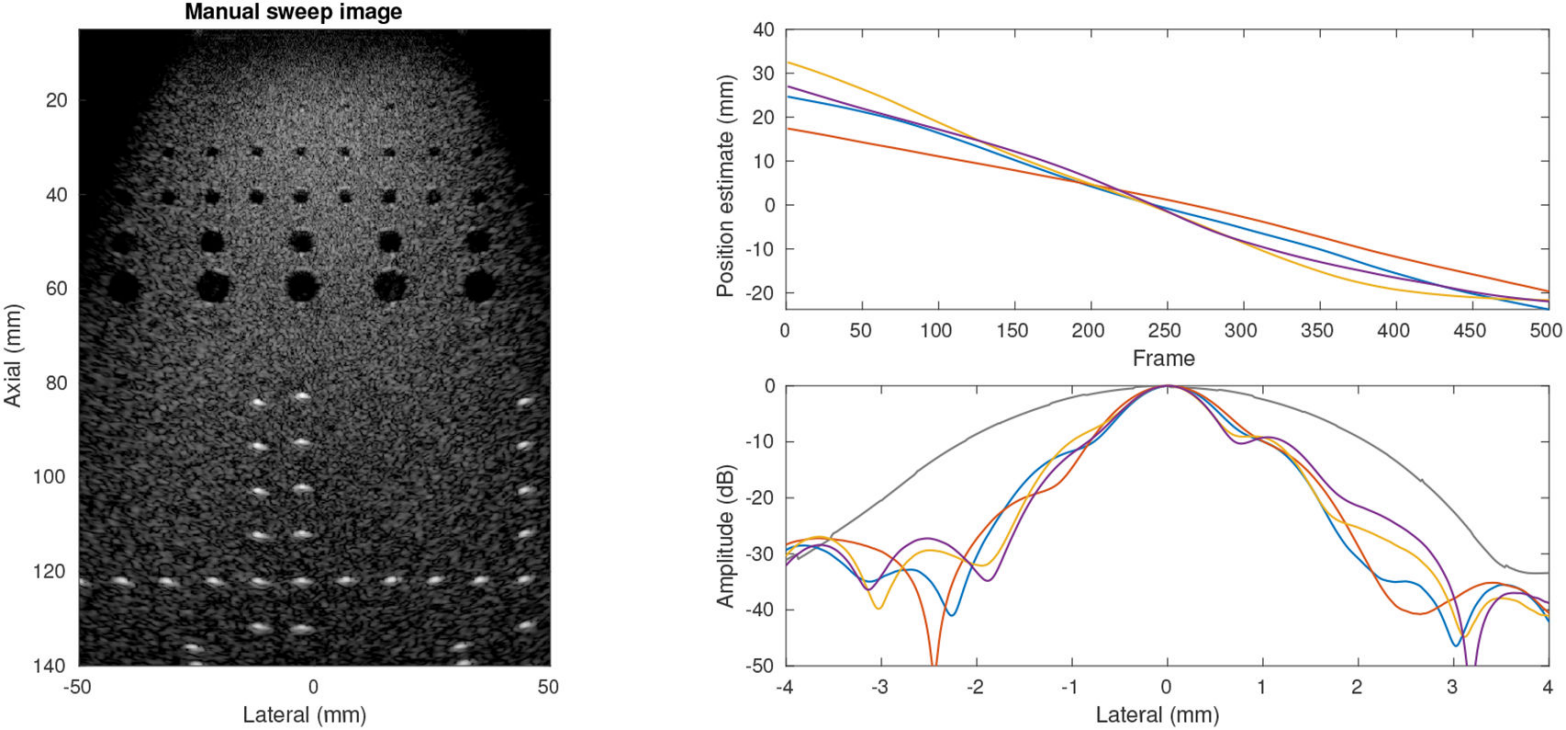
Results of four manual sweeps using linear slide fixture. (left) Sample SSA image from one manual sweep using channel correlation estimate (50 dB dynamic range). (right top) Motion estimates showing variation in sweep extent and temporal profile of manual sweeps. (right bottom) Lateral PSF for manual sweeps showing consistent results with slight variations due to phantom position and sweep extent. Reference PSF from Fig. 8 shown in black.

With an established baseline case showing that channel correlation and speckle tracking produce motion profiles that create SSA images equivalent to the ground truth for an ideal motion case, we studied the application of the channel correlation approach to a freehand sweep using the linear slide fixture. Fig. 10 shows four sweeps over approximately the same phantom region with varying temporal profiles and extents due to the manual motion. For image formation, these profiles were resampled using a fixed spatial sampling to avoid artifacts from varying sweep speeds, such as would be caused by the stationary region at the end of the yellow curve. We observed comparable performance across three cases with FWHM of 1.09 mm (54 mm sweep), 1.07 mm (49 mm sweep), and 1.14 mm (48 mm sweep), while the shortest sweep (37 mm) had a slightly larger FWHM of 1.30 mm.

**Figure 11.**
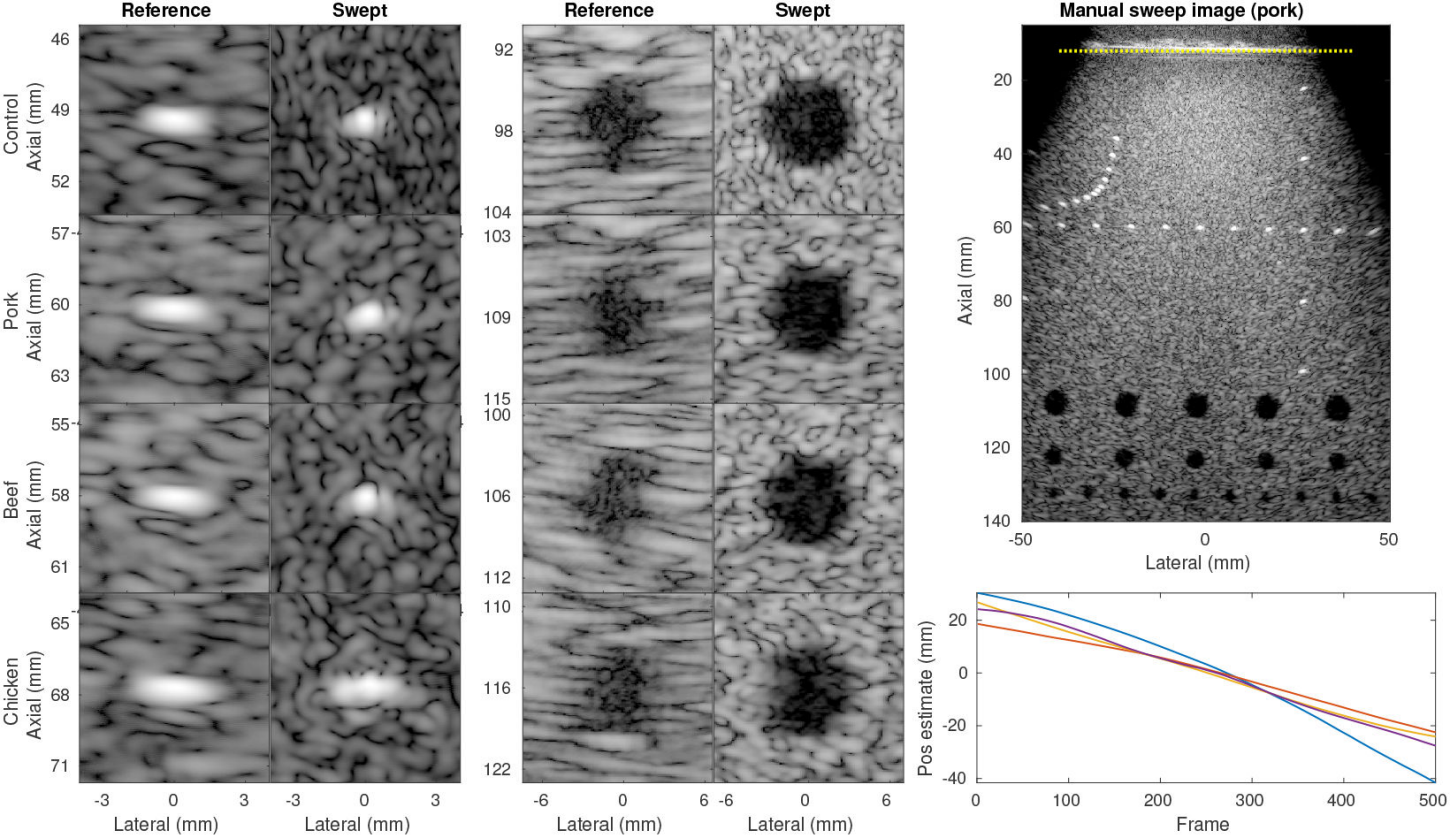
Results of manual sweeps through various tissue phantoms using linear slide fixture. All images show 50 dB dynamic range. (left and middle) Zoomed images of point and lesion targets from reference (no sweep) and manual sweep data. Spatial axes labels are approximate because position varies slightly between sweeps and targets. (right top) Sample SSA image through pork tissue (12 mm thick, above the dotted yellow line). (right bottom) Estimated positions for each case using channel estimation method.

Finally, we added an additional challenge to estimation by inserting an *ex vivo* tissue layer under between the transducer and phantom. Fig. 11 shows the results of the control case and imaging through the three different tissue layers (pork, beef, chicken). The FWHM improved from 1.47 mm to 0.59 mm, 1.76 mm to 0.83 mm, 1.73 mm to 0.67 mm, and 1.94 mm to 1.39 mm respectively. The control case had the point target at a smaller depth due to the lack of tissue layer and was expected to therefore have lower FWHM, and additionally had the longest sweep (72 mm vs 41 mm, 51 mm, and 52 mm respectively). Qualitatively, the tissue layers were found to add only small amounts of acoustic clutter compared to the control case, with the point target in the chicken breast case showing some broadening possibly due to aberration. The gCNR in the reference case was reduced from 0.82 to 0.70, 0.74, and 0.75 respectively by the tissue layers. These improved to 0.94, 0.93, 0.91, and 0.88 respectively using SSA imaging.

The large difference in FWHM after SSA imaging can be explained by the difficulty of performing the sweep procedure on the chicken breast layer. The tissue was slick, soft, and of varying thickness, making it very difficult to hold the fixture still on the surface while sweeping the transducer. The case presented is the best of six acquisitions, with the others showing severely degraded images (worse than the reference) due to fixture motion. This difficulty was not observed in the other tissue cases or control case, which all consistently produced high quality SSA results. However, the lesion results showed strong improvement in all cases, even in the chicken breast case despite the limited point target improvement. The gain in both SNR and overall resolution at depth due to SSA imaging greatly improved lesion detectability and image quality.

## 4. Discussion

The simulation and phantom results suggest that both speckle tracking and channel correlation are potential techniques for SSA imaging with a constrained sweep. However, there are distinct trade-offs in these algorithms. Channel tracking showed clear advantages in accuracy of estimation in these tasks and, importantly, greatly reduces the computational complexity of estimation. For example, in the phantom case of Fig. 7, using a Ryzen 7 2700X 8-core CPU, speckle tracking required 8.5 seconds to beamform the set of 501 image frames (246 x 164 pixels) used in the correlations and 13 seconds to perform the lateral cross-correlation search across frame pairs up to 10 frames apart and final weighted least squares estimate. The channel correlation approach took only 9 seconds total (for the cross-correlation search) using the same estimation parameters. In addition to avoiding the need for image formation, channel correlation uses a reduced spatial sampling because it is based on the aperture spacing rather than the sampling requirements in the imaging field. While we could reduce the lateral pixel spacing for speckle tracking closer to the Nyquist limit, the required sampling will depend on the depth of estimation. In both methods, more computation is required for larger the kernel and search regions. The above times should be used for comparison only, as GPU beamforming would improve image formation times and a more optimized implementation of normalized cross-correlation (e.g. re-using overlapping calculations, reducing for loops) would improve correlation times for both methods.

The largest challenge in this approach is any motion of the fixture that occurs outside of the imaging plane. These motions cannot be estimated using either tracking approach and degrade imaging performance as signals from across the sweep cannot be coherently combined. We demonstrated here that on relatively firm tissues (pork, beef) we were able to maintain the fixture position with sufficient accuracy to produce SSA images, comparable to the phantom alone. Future work will explore the use of these techniques *in vivo*, where different choices of target may present different scanning challenges. It may be possible, for example, to add an adhesive to the linear fixture if sliding is a problem, but if the tissue under the fixture compresses then the fixture may still tilt relative to the anatomy of interest. It may be beneficial to explore coupling the device to skin with a gel pad rather than gel, providing a firmer surface for the fixture and a more consistent coupling across the sweep. It will also be necessary to study the role that *in vivo* target movement (e.g. skin deformation, cardiac-induced motion, breathing) has on the SSA images even when the fixture is stationary relative to the skin. A sufficiently rapid sweep relative to the target motion could prevent degradation or adaptive motion filters may be required to process the data. This study was also limited in its analysis of acoustic clutter, which should be more directly assessed in more challenging imaging cases in the future.

SSA imaging introduces a new dimension of variability in image quality not present in conventional imaging. Even after normalizing for the spatial/temporal dynamics of the sweep, the sweep extent will result in varying effective resolution depending on the length. It may be desirable to limit the sweep length in post-processing to achieve more consistent results between acquisitions.

There are several computational challenges to address for clinical implementation of the constrained SSA approach. First, the current multi-lag displacement estimation approach used in this paper requires all images in the sequence before estimates are made and available for beamforming. A local estimator (or even estimates from individual image pairs) could be used instead so that beamforming could begin during data acquisition for more rapid results. Beamforming itself presents a challenge in that time delays need to be recalculated for each observed array position relative to the target pixel grid. We have already implemented a downsampling of acquired frames for beamforming – it may be possible to strategically choose these samples to align with precomputed array positions to avoid recalculating delays, although slight position error may result. Finally, over a one second sweep an operator will be left without live image guidance in the proposed approach. It should be possible to beamform the low-resolution frames for guidance or to interleave a short B-mode imaging sequence with the SSA transmissions to provide live guidance images while the high-resolution SSA image is completed.

If it were possible to make similar estimates in other dimensions, it may be possible to make SSA a truly freehand technique (i.e. without constrained motion). Estimates in the axial dimension are possible as in flow or elasticity imaging, and 2-D motion estimates are becoming more common using vector flow imaging techniques. However, more research is needed to translate these techniques to the problem of estimation transducer motion. Out-of-plane motion will remain a challenge unless using a matrix array, although we expect that the lower elevation resolution should also reduce the sensitivity to motion in that dimension.

## 5. Conclusions

We have demonstrated two signal processing approaches to transducer motion estimation within a constrained sweep that enable swept synthetic aperture imaging without external motion tracking. The constrained SSA motion problem is extremely well suited to high precision estimates given uniform motion of the field within the imaging plane, the ability to use large kernels for estimation, and high frame rate tracking. This approach may greatly reduce the cost and complexity of an SSA imaging system and is a step towards full freehand motion estimation. We plan to explore the use of this system for *in vivo* imaging to improve resolution and target conspicuity at depth without the need for large, expensive imaging arrays.

## Data Availability

The data presented in this study are available on request from the corresponding author. Code for speckle tracking and channel correlation using the weighted least squares approach, with a sample simulated data set, can be accessed at https://github.com/bottenuslab/lateral_transducer_tracking (DOI 10.5281/zenodo.7682384).

## Abbreviations

The following abbreviations are used in this manuscript:

FWHM: Full-width at half-maximum
gCNR: Generalized contrast-to-noise ratio
HCC: Hepatocellular carcinoma
PSF: Point spread function
RF: Radio frequency
ROI: Region of interest
SSA: Swept synthetic aperture
